# The effectiveness of exercise on cervical radiculopathy: A protocol for systematic review and meta-analysis

**DOI:** 10.1097/MD.0000000000016975

**Published:** 2019-08-30

**Authors:** Long Liang, Xin Cui, Minshan Feng, Shuaiqi Zhou, Xunlu Yin, Feng He, Kai Sun, He Yin, Rong Xie, Dian Zhang, You Zhou, Yue Wu, Guihong Tan, Zhengdong Wang, Xingyu Wang, Jianhua Zhang, Liguo Zhu, Jie Yu, Xu Wei

**Affiliations:** a Wangjing Hospital of China Academy of Chinese Medical Sciences; b Beijing Key Laboratory of Orthopedics of Traditional Chinese Medicine, Dongcheng District; c Beijing University of Chinese Medicine, Chaoyang District, Beijing; d First Affiliated Hospital of Anhui University of Chinese Medicine, Shushan District, Anhui, China.

**Keywords:** cervical radiculopathy, exercise, meta-analysis, protocol

## Abstract

**Background:**

Significant functional limitations and disabilities are common presenting complaints for people suffering from cervical radiculopathy. Exercise is a common conservative treatment for this disease. Therefore, we conducted a systematic review and meta-analysis to explore the efficacy of exercise in the treatment of cervical radiculopathy.

**Methods:**

A systematic literature search for studies will be performed in 7 databases, including PubMed, Web of Science, Embase, the Cochrane Library, the Chinese National Knowledge Infrastructure Database (CNKI), Wanfang database, and VIP database. The methodological quality of the included studies using the risk bias assessment tool of Cochrane and the level of evidence for results are assessed by the GRADE method. Statistical analysis is conducted with Revman 5.3.

**Results:**

This systematic review and meta-analysis will provide a synthesis of existed evidences for exercise on cervical radiculopathy.

**Conclusion:**

The conclusion of this study will provide evidence to assess effectiveness of exercise on cervical radiculopathy, which can further guide clinical decision-making.

**PROSPERO registration number:**

CRD42019121886

## 1 Introduction

Cervical radiculopathy, which is a normal result of degenerative changes such as creased disc height and zygapophyseal joints hyperplasia, is characterized by neck and shoulder pain with a combination of sensory loss or loss of motor function.^[1,2]^ According to the study conducted by Radhakrishnan et al,^[3]^ the annual age-adjusted incidence rate was 83.2 per 100,000 persons with a peak incidence in the fifth and sixth decade. From 2000 to 2009, the annual costs of anterior fusion in surgery methods has increased threefold ($1.62 billion to $5.63 billion).^[4]^ At present, treatment for cervical radiculopathy includes surgical and nonsurgical approaches, both of which aim to improve pain and nerve function, and prevent recurrence of cervical radiculopathy. Usually, surgical intervention should be considered for patients with radiographic characteristics of nerve compression with corresponding signs and symptoms, no improvement after 6 to 12 weeks of nonoperative treatment, or progressive neurological deficit.^[1,5]^ Most patients with cervical radiculopathy will improve with nonoperative care, although there are short of high-quality literature to support the results.^[6]^ Currently, conservative treatments are composed of pharmacotherapy, physical therapy, exercise, epidural steroid injections manipulation, ancillary treatments such as bracing, traction, acupuncture, and various combinations of these.^[7]^

Exercise, which contribute to pain and symptom relief, has gained popularity through promising results.^[8,9]^ Nevertheless, the studies evaluating physical exercise for patients with cervical radiculopathy are of various quality, and the number of patients in the trials is small. Moreover, the guidelines for exercise therapy for cervical radiculopathy have not clear recommendations or weak recommendations due to lacking of high quality literature.^[7,10–12]^ To the best of our knowledge, there is a systematic review on this topic at present,^[13]^ but it does not merge data for meta-analysis, which could not fully reflect the role of exercise in the treatment of cervical radiculopathy. So this systematic review and meta-analysis is to quantitative evaluate the existing literature about exercise therapy for cervical radiculopathy to help clinical decision-making.

## 2 Methods

This is a literature-based study, and thus no ethical approval and patient consent are required. The protocol of this study has been registered on the

International Prospective Register of Systematic Reviews (PROSPERO) (registration no.CRD42019121886, which is available on http://www.crd.york.ac.uk/PROSPERO/display_record.php?ID=CRD42019121886) basing on the Preferred Reporting Items for Systematic Reviews and Meta-Analyses Protocols (PRISMA-P) statement guidelines.^[14]^

### 2.1 Literature research

Relevant literature will be retrieved using multiple online databases including PubMed, Web of Science, Embase, the Cochrane Library, the Chinese National Knowledge Infrastructure Database (CNKI), Wanfang database, and VIP database. No limits are imposed on study date or publication language, type, and status. The key terms used in these searches are: “cervical radiculopathy,” “cervical spondylotic radiculopathy,” “nerve-root type cervical spondylosis,” “neck and arm pain,” “neck and shoulder pain,” “neck pain with radiculopathy,” “neck disorder with radiculopathy,” “exercise,” “motion,” “physical activity.” Different search strategies will be used for the Chinese and foreign language databases. In addition, the reference lists of previously published systematic reviews on the subject of exercise for the treatment of cervical radiculopathy are manually examined for pertinent studies.

### 2.2 Inclusion criteria

The retrieved literature is screened by 2 independent reviewers to evaluate eligibility, and any discrepancies are settled by discussion and consensus. First, the titles and abstracts of searched studies are screened. Then, full papers are reviewed to examine whether each study meets the following criteria: randomized controlled trial; type of participants must be patients with symptomatic diagnosed cervical radiculopathy according to the clinical and radiological criteria; experimental studies using exercise (including exercise only and other treatments with exercise). Control group is not restricted, but exercise is not included. When multiple time points were reported either in one particular report of a study or over the course of several articles from the same study, the longest follow-up period on treatment is considered in our article. If overlapping subject populations are enrolled in different reports, the one of higher quality or with a larger sample size will be selected for inclusion.

### 2.3 Exclusion criteria

The studies are excluded due to the following reasons: studies does not conform to the above criteria; both the treatment group and the control group included exercise; studies were in the form of letters, abstracts, reviews, or comments; studies are impossible to extract relevant data; the cervical radiculopathy patients were treated with surgery.

### 2.4 Data extraction

The following data are independently extracted by 2 authors via a purpose-designed form: the name of first author, year of publication, country, number of patients under exercise group and the control group, sample size, age, sex of patients, disease course, follow-up duration, outcome and intervention period. We will contact the authors by email or in other ways if the data are missing, wrong, or unclear. The authors resolve any disagreements by discussion, including input from a third author if required.

### 2.5 Quality assessment

We assessed the risk of bias of RCTs in this review using the Cochrane Collaboration Risk of Bias Tool.^[15]^ And risk of bias is assessed according to the Cochrane Handbook. For included study, types of bias are divided into 3 levels: low, unclear, high. Four authors independently assess the risk of bias of the included studies. The review authors resolve any disagreements by discussion, including input from a third independent review author if required.

### 2.6 Outcome measures

Visual analog scale (VAS), neck disability index (NDI), 36 (12)-Short Form Health Survey (SF-36 or SF-12), the 3 most recommended indicators in the guidelines—“An evidence-based clinical guideline for the diagnosis and treatment of cervical radiculopathy from degenerative disorders” issued by the North American Spine Society,^[7]^ are selected as outcomes. The primary outcome is VAS, and the secondary outcome are NDI and SF-36 (or SF-12).

### 2.7 Data synthesis and statistical analysis

The dichotomous data is expressed as the risk ratio (RR). And mean difference (MD) is used to assess the difference in the continuous outcomes between the groups, the confidence intervals (CI) for both dichotomous and continuous data. Statistical heterogeneity across the included studies will be examined using the *I*^2^ statistic, with an *I*^2^ >50% regarded as being indicative of the possibility of statistical heterogeneity, resulting in the selection of a random-effects model for merging of results. Otherwise, no obvious heterogeneity will be considered to be present in the included studies for values of *I*^2^ <50%, in which case the fixed-effects model will be selected. In order to evaluate the sensitivity of the meta-analysis, articles will be excluded one by one. The differences of the combining effects before and after exclusion will be compared. If the pooled outcomes are found to have been reversed after the exclusions, the outcomes may be unstable. Data regarding outcomes in the eligible trials are combined using the RevMan 5.3 software (Copenhagen, The Nordic Cochrane Centre, The Cochrane Collaboration, 2014), and the significance threshold will be a 2-sided *P* < .05.

### 2.8 Grade

The Grading of Recommendations Assessment, Development, and Evaluation (GRADE) method will be used to assess the quality of the evidence, which rate it into 4 levels: high, moderate, low, and very low.

## 3 Discussion

Rates of surgical procedures for cervical radiculopathy have grown rapidly, however, more and more attention has been paid to nonoperative management strategies due to the attendant costs and complication risks.^[16]^ Exercise is one of the important intervention method for this diseases.^[17]^ Exercise could increase neck flexor endurance and the cervical stabilization.^[17,18]^ In addition, Stolzman and Bement^[19]^ found exercise may activate conditioned pain modulation descending inhibitory pathways resulting in subsequent pain relief.

After careful literature search, we found there is no quantitative meta-analysis on the treatment of cervical radiculopathy by exercise so far. It is, therefore, necessary to carry out a study to assess the efficacy of exercise therapy for cervical radiculopathy. Furthermore, we hope the results of this study can help to propose the clinical recommendation for cervical radiculopathy and to provide more reliable evidence about the application of exercise therapy.

## Author contributions

**Conceptualization:** Long Liang, Xin Cui, Liguo Zhu.

**Data curation:** He Yin, Rong Xie, Xingyu Wang.

**Methodology:** Minshan Feng, Shuaiqi Zhou, Xunlu Yin, Feng He.

**Resources:** Kai Sun, He Yin, Rong Xie, Dian Zhang, You Zhou.

**Software:** Yue Wu, Guihong Tan, Zhengdong Wang, Xingyu Wang.

**Supervision:** Jianhua Zhang, Liguo Zhu, Jie Yu, Xu Wei.

**Writing – original draft:** Long Liang, Xin Cui, Shuaiqi Zhou, Xunlu Yin, Feng He.

**Writing – review & editing:** Long Liang, Minshan Feng, Liguo Zhu, Jie Yu, Xu Wei.

## References

[1] Carette S Fehlings MG Clinical practice. Cervical radiculopathy. N Engl J Med 2005;353:392–9. 16049211 10.1056/NEJMcp043887

[2] Corey DL Comeau D Cervical radiculopathy. Med Clin North Am 2014;98:791–9. xii. 24994052 10.1016/j.mcna.2014.04.001

[3] Radhakrishnan K Litchy WJ O’Fallon WM Epidemiology of cervical radiculopathy. A population-based study from Rochester, Minnesota, 1976 through 1990. Brain 1994;117 (pt):325–35. 8186959 10.1093/brain/117.2.325

[4] Alosh H Li D Riley LH Health care burden of anterior cervical spine surgery: national trends in hospital charges and length of stay, 2000–2009. J Spinal Disord Tech 2015;28:5–11. 24136049 10.1097/BSD.0000000000000001

[5] Onks CA Billy G Evaluation and treatment of cervical radiculopathy. Prim Care 2013;40:837–48. vii–viii. 24209721 10.1016/j.pop.2013.08.004

[6] Childress MA Becker BA Nonoperative management of cervical radiculopathy. Am Fam Physician 2016;93:746–54. 27175952

[7] Bono CM Ghiselli G Gilbert TJ An evidence-based clinical guideline for the diagnosis and treatment of cervical radiculopathy from degenerative disorders. Spine J 2011;11:64–72. 21168100 10.1016/j.spinee.2010.10.023

[8] Bertozzi L Gardenghi I Turoni F Effect of therapeutic exercise on pain and disability in the management of chronic nonspecific neck pain: systematic review and meta-analysis of randomized trials. Phys Ther 2013;93:1026–36. 23559524 10.2522/ptj.20120412

[9] Saal JS Saal JA Yurth EF Nonoperative management of herniated cervical intervertebral disc with radiculopathy. Spine (Phila Pa 1976) 1996;21:1877–83. 8875719 10.1097/00007632-199608150-00008

[10] Cohen SP Hooten WM Advances in the diagnosis and management of neck pain. BMJ 2017;358:j3221. 28807894 10.1136/bmj.j3221

[11] Kjaer P Kongsted A Hartvigsen J National clinical guidelines for non-surgical treatment of patients with recent onset neck pain or cervical radiculopathy. Eur Spine J 2017;26:2242–57. 28523381 10.1007/s00586-017-5121-8

[12] Leveque JC Marong-Ceesay B Cooper T Diagnosis and treatment of cervical radiculopathy and myelopathy. Phys Med Rehabil Clin N Am 2015;26:491–511. 26231961 10.1016/j.pmr.2015.04.008

[13] Cheng CH Tsai LC Chung HC Exercise training for non-operative and post-operative patient with cervical radiculopathy: a literature review. J Phys Ther Sci 2015;27:3011–8. 26504347 10.1589/jpts.27.3011 PMC4616148

[14] Shamseer L Moher D Clarke M Preferred reporting items for systematic review and meta-analysis protocols (PRISMA-P) 2015: elaboration and explanation. BMJ 2015;350:g7647. 25555855 10.1136/bmj.g7647

[15] Higgins JP Altman DG Gotzsche PC The Cochrane Collaboration's tool for assessing risk of bias in randomised trials. BMJ 2011;343:d5928. 22008217 10.1136/bmj.d5928 PMC3196245

[16] Fritz JM Thackeray A Brennan GP Exercise only, exercise with mechanical traction, or exercise with over-door traction for patients with cervical radiculopathy, with or without consideration of status on a previously described subgrouping rule: a randomized clinical trial. J Orthop Sports Phys Ther 2014;44:45–57. 24405257 10.2519/jospt.2014.5065

[17] Akkan H Gelecek N The effect of stabilization exercise training on pain and functional status in patients with cervical radiculopathy. J Back Musculoskelet Rehabil 2018;31:247–52. 28946516 10.3233/BMR-169583

[18] Halvorsen M Falla D Gizzi L Short- and long-term effects of exercise on neck muscle function in cervical radiculopathy: a randomized clinical trial. J Rehabil Med 2016;48:696–704. 27494094 10.2340/16501977-2120

[19] Stolzman S Bement MH Does exercise decrease pain via conditioned pain modulation in adolescents? Pediatr Phys Ther 2016;28:470–3. 27661245 10.1097/PEP.0000000000000312 PMC5098832

